# Effective harvesting, detection, and conversion of IR radiation due to quantum dots with built-in charge

**DOI:** 10.1186/1556-276X-6-584

**Published:** 2011-11-07

**Authors:** Kimberly Sablon, Andrei Sergeev, Nizami Vagidov, Andrei Antipov, John Little, Vladimir Mitin

**Affiliations:** 1 U.S. Army Research Laboratory, Powder Mill Road, Adelphi, MD, 20783-1197, USA; 2 University at Buffalo, State University of New York, Buffalo, NY, 14260-1920, USA

**Keywords:** quantum dot, infrared photodetector, solar cell, photoresponse, doping, potential barrier, capture processes

## Abstract

We analyze the effect of doping on photoelectron kinetics in quantum dot [QD] structures and find two strong effects of the built-in-dot charge. First, the built-in-dot charge enhances the infrared [IR] transitions in QD structures. This effect significantly increases electron coupling to IR radiation and improves harvesting of the IR power in QD solar cells. Second, the built-in charge creates potential barriers around dots, and these barriers strongly suppress capture processes for photocarriers of the same sign as the built-in-dot charge. The second effect exponentially increases the photoelectron lifetime in unipolar devices, such as IR photodetectors. In bipolar devices, such as solar cells, the solar radiation creates the built-in-dot charge that equates the electron and hole capture rates. By providing additional charge to QDs, the appropriate doping can significantly suppress the capture and recombination processes via QDs. These improvements of IR absorption and photocarrier kinetics radically increase the responsivity of IR photodetectors and photovoltaic efficiency of QD solar cells.

## Introduction

One of the main goals for the next generation of infrared [IR] imaging systems and solar cell photovoltaic devices is to increase the photoresponse to IR radiation [1]. To enhance the IR photoresponse, it is necessary to (1) improve electron coupling to IR radiation and (2) increase the photocarrier lifetime, i.e., to suppress recombination losses. However, it is not easy to increase IR absorption without enhancement of recombination losses because by introducing electron levels, which provide strong IR transitions, we inevitably create additional channels for inverse processes that increase recombination losses.

This trade-off between IR absorption and recombination processes are well understood for a number of technologies and corresponding materials. For example, in the early 1960s, semiconductors with impurities which provide electron levels inside a semiconductor bandgap and induce IR transitions from localized impurity states to conducting states received significant attention. However, midgap impurities drastically enhance the recombination processes, i.e., the Shockley-Read-Hall recombination, and deteriorate the photovoltaic conversion efficiency [2,3].

To accommodate the solar spectrum and utilize its IR portion, modern photovoltaic technology mainly employs multi-junction cells with different bandgaps [4]. In these devices, each p-n junction cell is designed to effectively harvest solar energy within a certain spectral window close to the bandgap. According to theoretical modeling, in a multi-junction solar cell with five or more junctions, the ultimate photovoltaic efficiency may exceed 70%. However, current technology enables only triple-junction cells (Ge-substrate junction-InGaAs-AlInGaP) with the maximum conversion efficiency of approximately 42% for concentrator cells. Strong technological limitations are caused by the need for lattice match, thermal expansion match, and current match in the cascade of heterojunctions [5,6].

Quantum-well structures are intensively investigated for applications in IR imaging and solar energy conversion. Some enhancement in conversion efficiency was observed in solar cells, based on planar quantum wells, due to increased resonance absorption. Quantum-well IR sensing is currently a well-established technology, which is widely used for detection and imaging at liquid nitrogen temperatures and below. However, at higher temperatures, the photoresponse tremendously decreases due to a strong reduction of photocarrier lifetime.

Recently, quantum-dot [QD] structures have attracted much attention due to their ability to enhance absorption of IR radiation via multiple energy levels introduced by QDs [7-9]. In QDs, the carriers are confined in all three dimensions. Electron states in separate dots can be connected via manageable tunneling coupling between QDs. Therefore, QD media provide numerous possibilities for nanoscale engineering of electron spectra by varying the dot size and shape as well as the concentration of QDs and geometry of a QD structure. Besides tunable IR absorption, QD structures offer wide flexibility for nano-engineering of electron processes via the built-in-dot charge, correlation of dot positions, and selective doping. The built-in charge induced by selective doping creates potential barriers around dots and prevents capture of carriers of the same sign as the built-in-dot charge.

In very recent works, we have reported a radical improvement on the responsivity of QD infrared photodetectors [QDIP] [10] and QD solar cell efficiency [11] due to strong inter-dot doping, which creates substantial built-in-dot charge. While up to now the incorporation of QDs improves the solar cell's performance just by a few percent [12], we demonstrated that QDs with the built-in charge of approximately six electrons per dot provide a 50% increase in photovoltaic efficiency [11]. We also observed approximately 25 times increase of the photoresponse of QDIP when the built-in-dot charge increases from one electron to six electrons per dot [10]. Research on the capabilities of QD media with built-in-dot charge is still far from completion.

In this work, we investigate the physical processes behind these radical improvements. We study the potential relief created by the built-in-dot charge and calculate potential barriers, which separate the conducting states in the media from the localized QD states. Taking into account the effects of the built-in-dot charge on the IR absorption and photoelectron kinetics, we propose a simple model, which adequately describes effects of doping on the operation of unipolar optoelectronic QD devices, such as QDIPs. We also analyze our data related to the operation of a QD solar cell and present basic contours of the model for the description of doping-induced effects in the kinetics of bipolar photocarriers in QD structures. We conclude that in both cases, the built-in-dot charge strongly enhances electron coupling to electromagnetic radiation and suppresses the most effective capture processes. These two factors allow us to improve the performance of QDIPs and QD solar cells.

### Unipolar kinetics in QD structures: IR photodetectors

**Figure 1:**
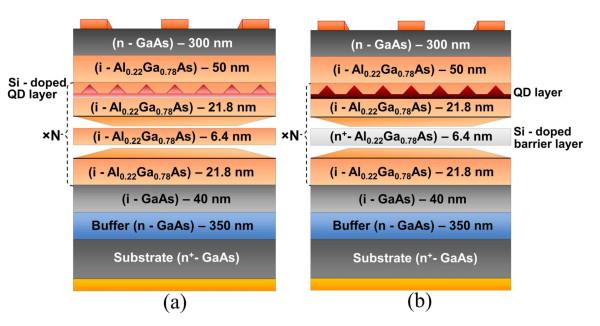
**QD structures**. QD structures with intra-dot doping, i.e., doping of QD layers (**a**) and inter-dot doping, i.e., doping between QD layers (**b**).

To investigate the effects of the built-in-dot charge on the unipolar kinetics in QD photodetectors, we investigate anisotropic potential barriers in real QD structures used for IR sensing. Our QD structures have been fabricated using molecular beam epitaxy with growth temperatures of 500 ± 10°C. InAs dots were grown on AlGaAs surfaces by deposition of approximately 2.1 monolayers of InAs. During the normal growth of layers, the substrate was rotated at 30 RPM to insure the uniform thickness of the layers. The thickness of GaAs spacer between the QD layers was chosen large enough to minimize the strain. The obtained structures were doped in two different ways: with intra-dot doping (devices B44 and B52) and with inter-dot doping (devices B45 and B53). In devices B44 and B52 (Figure 1a), the dopant sheet concentration was 2.7 × 10^11 ^cm^-2 ^and 5.4 × 10^11 ^cm^-2^, respectively. Devices B45 and B53 have been grown with Si dopants directly in the middle of each AlGaAs barrier layer (Figure 1b). In devices B45 and B53, the dopant sheet concentration was also 2.7 × 10^11 ^cm^-2 ^and 5.4 × 10^11 ^cm^-2^, respectively. QDs had the truncated pyramid shape with an average of 3.6 nm in height and 15 nm in width. The QDs were randomly distributed over the QD layer. The average distance between dots was 31 nm which corresponds to the sheet concentration of 10^11 ^cm^-2^. Parameters of our samples are summarized in Table 1. Details of the fabrication technique and other parameters of these devices may be found in Mitin et al. [10].

**Table 1:** QDIP devices

Device	B44	B45	B52	B53
Doping	Intra-dot	Inter-dot	Intra-dot	Inter-dot
Donor concentration (10^11 ^cm^-2^)	2.7	2.7	5.4	5.4
Number of electrons in dot, *n*	2.7	2.8	4.7	6.1
Built-in-dot charge, *n*_q_	1.8	2.8	3.45	6.1

To calculate the built-in-dot charge and investigate the potential profiles around dots, we used the nextnano^3 ^software, which allows for simulation of multilayer structures combined with different materials of realistic geometries in one, two, and three spatial dimensions [13]. This simulation tool self-consistently solves Schrödinger, Poisson, and current equations for electrons and holes. The conduction and valence bands of the structures are defined within a single-band or multi-band k·p model, which includes a strain.

**Figure 2:**
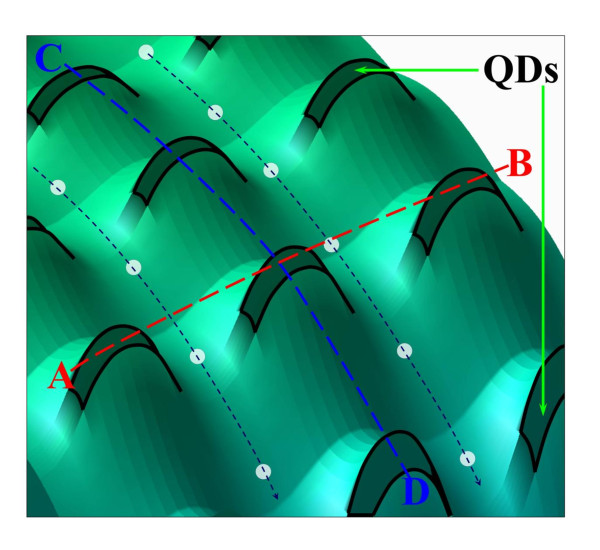
**3-D profile of potential barriers around dots with built-in charge**. A-B cross section is along the QD plane, and C-D cross section is in the direction of the vertical electron transport.

The three-dimensional [3-D] potential profile in QD structures calculated with nextnano^3 ^is shown in Figure 2. The light black lines denote the preferable channels for the motion of photoelectrons (white dots) in the potential relief created by the built-in-dot charge.

**Figure 3:**
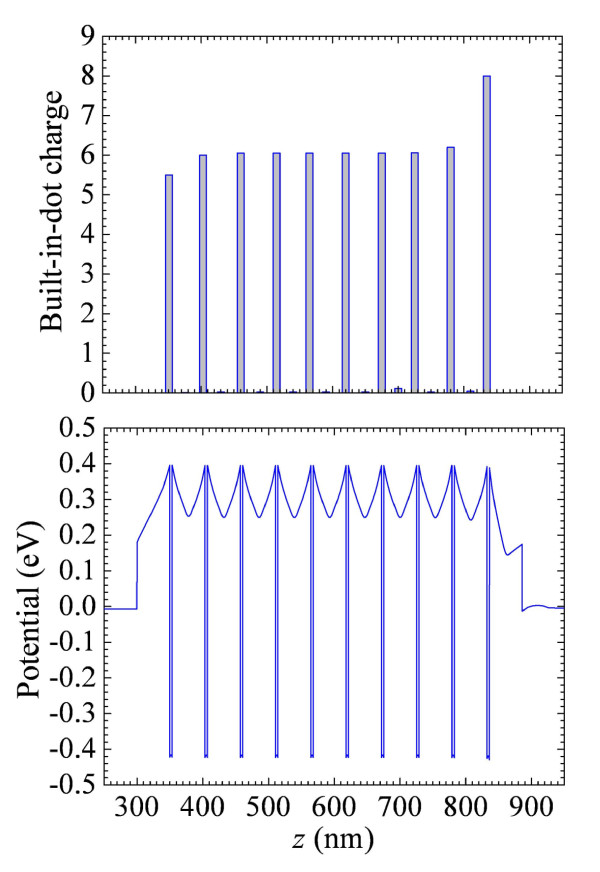
**Built-in-dot charge and potential distribution over the sample with ten QD layers**.

We simulated the band structure and potential distribution in real devices taking into account the effects of contacts. Figure 3 shows variations of the built-in-dot charge and potential profile in the C-D cross section for sample B53 with inter-dot doping (for clarity, we present it in ten QD layers). As seen, the effect of contacts is important only for one or two QD layers adjacent to the contacts. Thus, the built-in-dot charge in QD layers from the third to the eighth is directly determined by the inter-dot doping. In Table 1, we present the built-in-dot charge, which is determined by the number of captured electrons and number of dopants (in the case of intra-dot doping).

As seen in Figure 2, the potential barriers around QDs are strongly asymmetric. The barriers in the QD planes, i.e., in the direction perpendicular to the current, are substantially smaller than the barriers in the direction of the current. This asymmetry has strong consequences for the kinetics of photocarriers.

**Figure 4:**
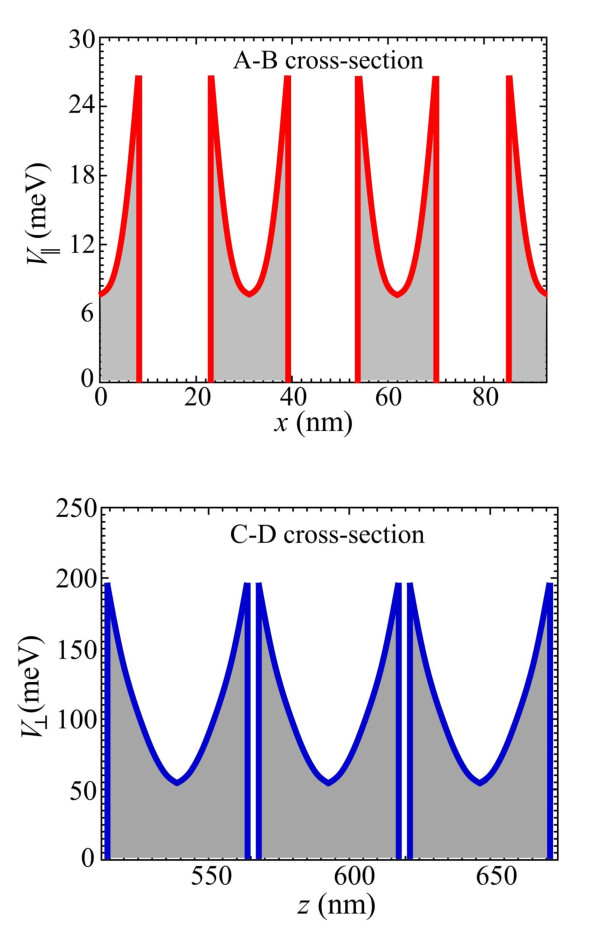
**Potential barriers**. Potential barriers around dots at the center of the QD structure in the A-B cross section (*x*-axis) and in the C-D cross section (*z*-axis).

In Figure 4, we compare the potential profiles in the A-B cross section (*x*-axis) and in the C-D cross section (*z*-axis). Potential barriers in the A-B cross section are significantly smaller, and therefore, they are presented with a higher resolution.

**Figure 5:**
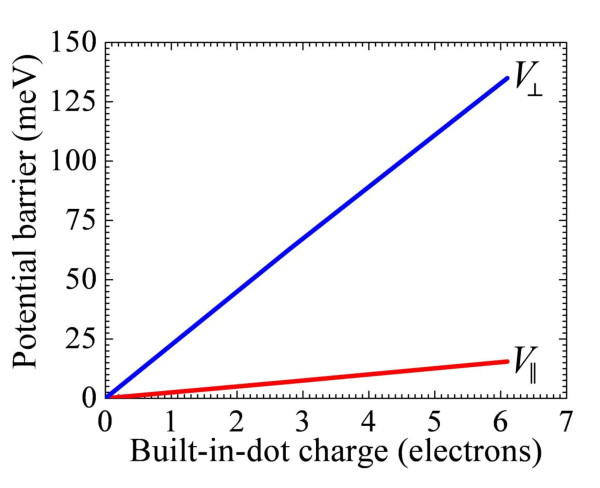
**Height of potential barriers around single dots in directions perpendicular and parallel to QD planes**.

Using the nextnano^3^, we have analyzed the local potential barriers around single QDs as a function of the built-in charge. As expected, these local barriers are independent on the QD position in the device and are strongly asymmetric because of the asymmetry of the QD shape. Figure 5 shows the height of local potential barriers around single dots in directions perpendicular and parallel to the QD planes as a function of the built-in-dot charge. Linear character of dependences is expected. In Figure 5, we highlight the strong anisotropy of the barriers, which is critically important for capture processes and photoelectron kinetics.

The photoelectron capture into the charged dot may be realized either via tunneling through the barrier or via thermal excitation above the barrier. The relative probability of these two processes depends on the characteristic size of the dot [14]. At room temperature, if a dot radius is smaller than approximately 5 nm, the electron capture by the dot is analogous to the capture by the repulsive impurity [15]. In this case, the capture rate is proportional to exp[-(*k*_B_*T/E*_B_)^1/3^], where *E*_B _is Bohr's energy; *E*_B _= 2*π*^2^*n*^2^e^4^*m*/*h*^2^*κ*, where *n *is the number of electrons captured in a dot, *m *is the electron mass, and *κ *is the permittivity [15]. In the opposite case, which is usually realized in QD structures, the thermally activated processes dominate over tunneling and the capture rate follows the exponential dependence [14,16,17]:

(1) $$\frac{1}{\tau_{\text{capt}}}\propto \exp\left[-\frac{V\left(Q\right)}{k_{\text{B}}T}\right],$$

where *V(Q) *is the height of the local potential barrier, which is a function of the built-in-dot charge *Q = en*_q_.

As shown in Figure 5, the height of the potential barrier in the direction parallel to the QD plane is substantially smaller than that in the perpendicular direction. Therefore, we expect that the capture processes in QD planes will dominate in the relaxation processes. Based on Figure 5, the corresponding barrier height is *V*_|| _= *bn*_q_, where *b *= 2.5 meV. In the case of the intra-dot doping, the dot charge *n*_q _is equal to the dot population *n *reduced by the number of dopants *p *in the dot, i.e., *n*_q _= *n - p*. In the case of the inter-dot doping, the built-in-dot charge *q *is obviously equal to *n*.

Thus, based on the above consideration, we expect that the effects of doping on the photocurrent in QD structures are described by:

(2) $$I=A\;n\exp\left(\frac{bn_{\text{q}}}{k_{\text{B}}T}\right)\;.$$

Here, *A *is some constant which does not depend on doping. The pre-exponential factor in Equation 2 describes the increase of the absorption with increasing number of electrons in the dot *n*. The exponential factor describes the effect of potential barriers around dots on the photoelectron lifetime. It is proportional to the dot charge *n*_q _determined by the number of electrons and number of dopants in the dot.

**Figure 6:**
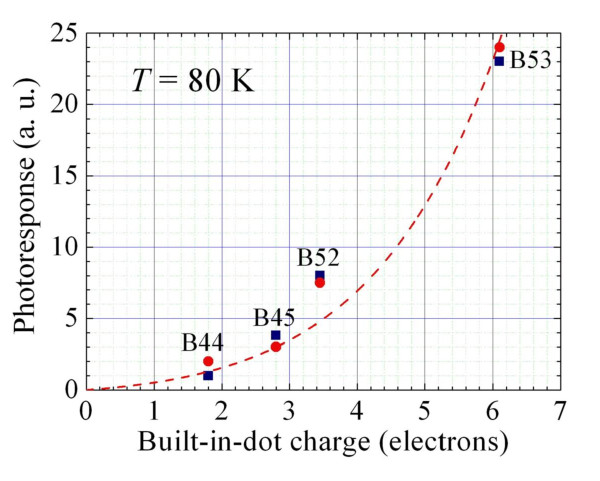
**The photocurrent as a function of the built-in-dot charge**. The blue squares are for experimental data and the red circles are for modeling results. The red dashed line is the theoretical dependence for the inter-dot doping.

In Figure 6, we apply the analysis of our experimental data, obtained from Mitin et al. [10], in the framework of this model. For fitting of our experimental results, we take values of *n*, determined from self-consistent modeling of potential profile using nextnano^3 ^(see Table 1). As seen, the theoretical modeling (red circles) is in a very good agreement with the experimental data (blue squares). From this fitting, the parameter *b *was found to be equal to 2.7 meV, which is in a good agreement with *b *= 2.5 meV that we obtained from the independent modeling of the potential barrier heights (see Figure 5). The red dashed line shows the modeling results for the inter-dot doping (*n = n*_q_), which was used for samples B45 and B53. For samples B44 and B52 with the doping of QD layers, the dot charge was formed by the electrons captured in the dot and dopants placed in the dot. In this case, *n = n*_q _*+ p *and the corresponding red circles are above the dashed line.

Thus, the proposed relatively simple model provides a very good description of doping effects on the photoresponse of QD structures. We believe that such good agreement with the experiment evidences that the model adequately takes into account the main effects of doping on photoelectron kinetics.

### Bipolar kinetics: solar cell with built-in-dot charge

The heterostructure solar cells are presently dominating the market of high-efficiency solar cells. They have a conversion efficiency of up to 42%, have high degradation robustness (enables applications in outer space), and allow for high concentration of solar energy. Despite the impressive achievements in heterostructure technologies, the pace of improvement of solar cell efficiency is very slow. It is limited by the following factors: thermalization losses, losses related to junction and contact voltages, and recombination losses. Multi-junction solar cells with different bandgaps have been developed to minimize thermalization losses in heterostructure solar cells. In these devices, each p-n junction cell is designed to effectively harvest solar energy within a certain spectral window close to the bandgap. To date, the triple-junction cells reach a maximum conversion efficiency of approximately 42%, in the case of concentrator cells. Technological limitations are determined by the need to match crystalline lattices, thermal expansion coefficients, and the most difficult, to match all the photoinduced currents in the cascade of heterojunctions.

QD structures are considered very promising photovoltaic nanomaterials due to their ability to extend the conversion of solar energy into the IR range [7-9]. Up to now, most of the emphasis has been placed on the QD solar cell with an intermediate band, which is formed from discrete QD levels due to tunneling coupling between QDs. Theoretical calculations predict that the intermediate-band solar cell can provide an efficiency of approximately 63%. However, intensive experimental efforts to improve the performance of intermediate-band solar cells show limited success. In comparison with a reference cell, the short-circuit photocurrent of the QD intermediate-band cells increases only by a few percent [12]. It is well understood that the addition of QDs significantly increases the absorption of IR radiation, but simultaneously, QDs drastically increase recombination processes. For this reason, the corresponding recombination losses are hardly compensated by the conversion of IR radiation. To solve this problem, one should further suppress the photocarrier capture into QDs.

As we have discussed in the previous section in relation to QDIP, potential barriers around dots provide an effective and reliable way to control the photoelectron processes at room temperatures. However, the bipolar kinetics of electrons and holes in QD structures is much more complex. The built-in-dot charge suppresses solely the capture processes of the carriers of the same sign as the dot charge. Again, this suppression is strong and has an exponential dependence on the dot charge (Equation 1). Under radiation, in stationary conditions of the dynamic equilibrium, the built-in-dot charge equates the capture rates of electrons and holes. Thus, to minimize recombination losses, the built-in-dot charge should be used for the suppression of the most effective capture processes. Here, we investigate this concept and study the effects of built-in-dot charge on IR harvesting, recombination, and efficiency of QD solar cells.

**Figure 7:**
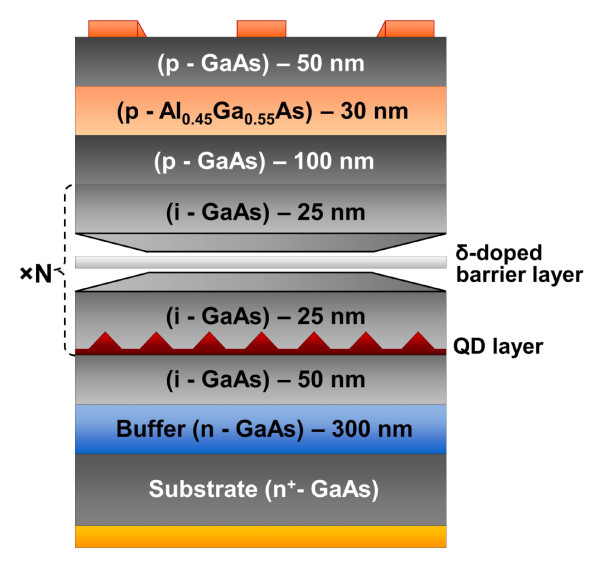
**A schematic layout of a *δ*-doped QD solar cell**.

For the experimental verification of our suggestions, we fabricated and investigated p- and n-doped InAs/GaAs QD solar cells with various doping levels. Figure 7 illustrates a typical solar cell with a modulation *δ-*doped QD structure in which a plane of dopants is placed in the middle of each GaAs layer that separates QD layers. These structures contain 20 stacks of InAs QD layers separated by GaAs with various dopant sheet densities providing zero, two, three, four, and six electrons per QD.

The effect of the built-in-dot charge on the capture processes has been studied by employing photoluminescence [PL] measurements. PL in QD solar cells was measured under short-circuit conditions. To stimulate PL, we used the 514-nm line from an Argon-ion laser. PL signals from the samples were measured by an InGaAs detector array.

**Figure 8:**
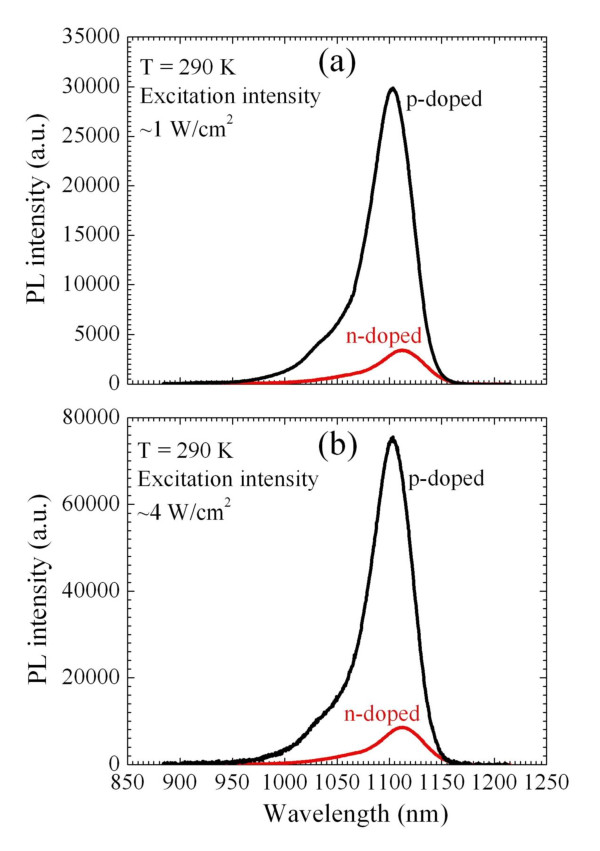
**Comparison of PL spectral dependences**. Comparison of PL spectral dependences of n- and p-doped samples with four carriers per dot under an intensity of (**a**) approximately 1 W/cm^2 ^and (**b**) approximately 4 W/cm^2^.

In Figure 8, we compare the PL from p- and n-doped samples (four carriers per dot) at 1- and 4-W/cm^2 ^intensities. As seen, p-doping drastically increases PL, which is realized via recombination processes in QDs. In n-doped devices, the PL intensity turns out to be approximately eight times weaker than that in p-doped devices. Therefore, based on our previous analysis, n-doping should suppress the fast electron-capture processes, minimize the recombination losses, and increase the solar cell performance, while p-doping is expected to deteriorate the photovoltaic efficiency. In other words, to effectively contribute to the photovoltaic conversion, an electron and a hole should simultaneously escape from the dot. The energy-level spacing for electrons in QDs is relatively large. It substantially exceeds the spacing for holes and thermal energy. For this reason, it is precisely the electron intra-dot processes which limit the electron-hole escape from QDs. Thus, it is critically important to enhance the photoexcitation of electrons rather than holes and at the same time, to suppress the electron-capture processes.

**Figure 9:**
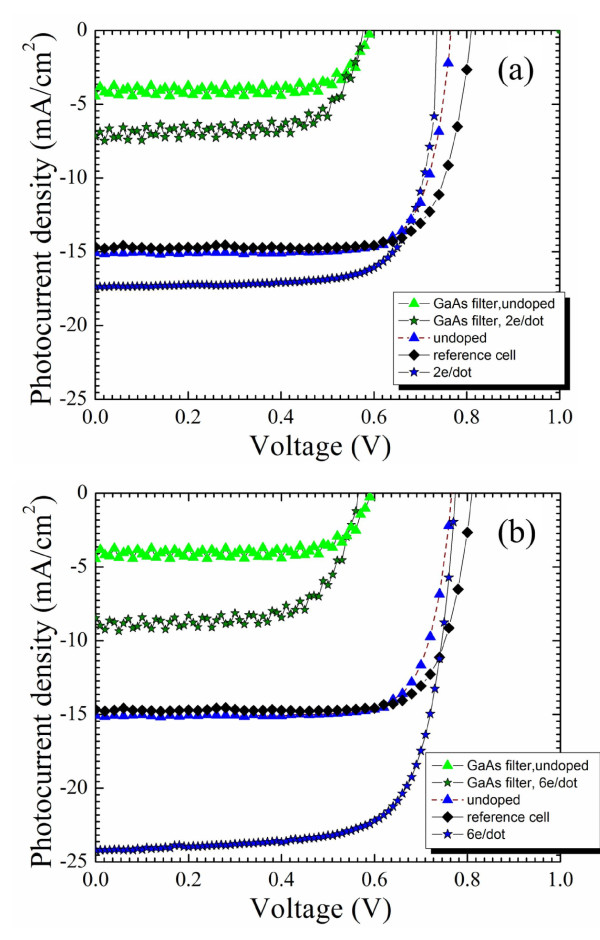
***I-V *characteristics of solar cells**. *I-V *characteristics of solar cells with built-in-dot charge of two electrons per dot (**a**) and six electrons per dot (**b**) in comparison with the reference cell (without QDs) and undoped QD solar cell.

Efficiency of the photovoltaic conversion in solar cell devices with the built-in-dot charge has been measured using a calibrated solar simulator. The corresponding *I-V *curves for devices with a built-in-dot charge of two and six electrons under 1 Sun (AM1.5G) irradiation are presented in Figures 9a, b, respectively. For comparison, in Figure 9, we also presented *I-V *curves for the reference cell without QDs and for the undoped QD solar cell. As seen, the short-circuit current increases with doping from approximately 15 mA/cm^2 ^in the reference cell and undoped QD cell to 17.5 mA/cm^2 ^for the device with two electrons per dot and further, to 24 mA/cm^2 ^for the device with six electrons per dot. As with the conventional solar cell with a p-n junction, doping also prevents deterioration of the open-circuit voltage.

The IR harvesting and conversion have been investigated by measurements of the photoresponse under 1 Sun radiation with the GaAs filter which eliminates high-energy photons with a wavelength less than 880 nm. *I*-*V *characteristics obtained with the GaAs filter are corrected for reflectivity losses and presented in Figure 9. As seen, the IR photoresponse significantly increases due to the built-in-dot charge. In the device doped to provide two electrons per dot, we observe an increase in the photocurrent of approximately 7.0 mA/cm^2 ^compared with the reference cell. The photocurrent from the sample with six electrons per dot increases by approximately 9 mA/cm^2^. As expected, the GaAs reference cell does not show any IR photoresponse. These measurements directly demonstrate strong harvesting and effective conversion of IR radiation by solar cells with the built-in-dot charge.

**Figure 10:**
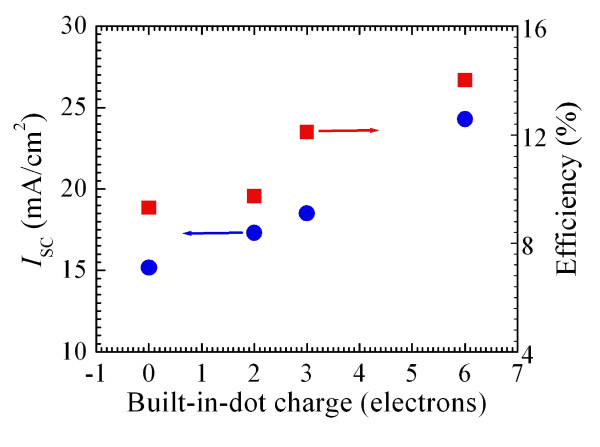
**Short-circuit current and efficiency of solar cells as a function of the built-in-dot charge**.

The basic parameters of our devices with the built-in charge of two, three, and six electrons per dot are summarized in Figure 10. As seen, the photovoltaic efficiency radically improves due to the built-in-dot charge. We have not observed any evidence of saturation of the effect, and therefore, even higher efficiencies are anticipated for higher doping. It should be noted that as it is done in other research projects [12,18-21], to minimize the cost, we fabricate test structures, which do not have antireflection coating and back surface field and are also relatively short (approximately 1.4 μm). As a result, the efficiency of our reference cell (without QDs) is less than the record efficiencies of GaAs solar cells (26% under unconcentrated light and 29% under concentrated light). However, the presented data provide strong evidence that QDs with built-in charge can significantly increase IR harvesting and conversion of light.

## Conclusions

Our new approach, based on engineering 3-D potential barriers introduced by QDs with built-in-dot charge, provides real opportunities to radically improve the performance of IR photodetectors and solar cells. These improvements are due to effective harvesting of IR radiation via intra-band QD transitions and transitions from QDs to the conducting states in the matrix. Potential barriers also prevent photoelectron capture and increase the photoelectron lifetime. Modern technologies allow for fabrication of specific structures with various positions/distributions of QDs. This provides numerous possibilities for engineering very specific 3-D barriers. We believe that in future electronic and sensing technologies, the 3-D barriers will be employed in every device as nowadays, technology employs semiconductor heterostructures with one-dimensional barriers.

## Competing interests

The authors declare that they have no competing interests.

## Authors' contributions

All authors planned the research and participated in the preparation of the paper. KS performed solar cell measurements and analyzed the experimental data. AS and NV developed the model and performed the corresponding simulations. AA made measurements related to quantum-dot infrared photodetectors. JL and VM conducted the design and analysis of all experiments and modeling results. All authors read and approved the final manuscript.
